# Is Neuroscience FAIR? A Call for Collaborative Standardisation of Neuroscience Data

**DOI:** 10.1007/s12021-021-09557-0

**Published:** 2022-01-21

**Authors:** Jean-Baptiste Poline, David N. Kennedy, Friedrich T. Sommer, Giorgio A. Ascoli, David C. Van Essen, Adam R. Ferguson, Jeffrey S. Grethe, Michael J. Hawrylycz, Paul M. Thompson, Russell A. Poldrack, Satrajit S. Ghosh, David B. Keator, Thomas L. Athey, Joshua T. Vogelstein, Helen S. Mayberg, Maryann E. Martone

**Affiliations:** 1 Faculty of Medicine, Jr. Brain Imaging Center, McGill UniversityMontreal Canada and Henry H. WheelerHelen Wills Neuroscience Institute, University of California Berkeley, Berkeley, CA USA; 2 Department of Psychiatry, University of Massachusetts Chan Medical School, Worcester, MA USA; 3 Redwood Center for Theoretical Neuroscience, University of California, Berkeley, CA USA; 4 Bioengineering Department, Neuroscience Program, and Center for Neural Informatics, Structures, and Plasticity; Volgenau School of Engineering and Krasnow Institute for Advanced Study, George Mason University, Fairfax, VA USA; 5 Anatomy & Neurobiology Department, Washington University in St. Louis, St. Louis, MO USA; 6 Brain and Spinal Injury Center, UCSF, University of California San Francisco School of Medicine, San Francisco, CA USA; 7 Center for Research in Biological Systems, University of California, San Diego, CA USA; 8 Allen Institute for Brain Science, Seattle, WA USA; 9 Imaging Genetics Center, Stevens Institute for Neuroimaging & Informatics, Keck School of Medicine, University of Southern California, Los Angeles, CA USA; 10 Department of Psychology and Stanford Center for Reproducible Neuroscience, Stanford University, Stanford, CA 94305 USA; 11 Massachusetts Institute of Technology, Boston, MA USA; 12 University of California Irvine, Irvine, CA USA; 13 Center for Imaging Science, Department of Biomedical Engineering, Johns Hopkins University, Maryland Baltimore, USA; 14 Institute for Computational Medicine, Center for Imaging Science, Department of Biomedical Engineering, Johns Hopkins University, Baltimore, Maryland USA; 15 Departments of Neurology and Neurosurgery, Icahn School of Medicine at Mount Sinai, New York, NY USA; 16 Department of Neurosciences, University of California, San Diego, La Jolla, CA 92093-0662 USA

**Keywords:** Neuroscience, Standards,, Interoperability, International Neuroinformatics Coordinating Facility

## Abstract

In this perspective article, we consider the critical issue of data and other research object standardisation and, specifically, how international collaboration, and organizations such as the *International Neuroinformatics Coordinating Facility (INCF)* can encourage that emerging neuroscience data be *Findable, Accessible, Interoperable, and Reusable (FAIR)*. As neuroscientists engaged in the sharing and integration of multi-modal and multiscale data, we see the current insufficiency of standards as a major impediment in the Interoperability and Reusability of research results. We call for increased international collaborative standardisation of neuroscience data to foster integration and efficient reuse of research objects.

As experimental assays and analyses become increasingly complex and the scale of tissue and cellular profiling multiplies, neuroscience faces increasing challenges. To be used efficiently, accessible data need to be described coherently and with standards. We present here the argument that standardisation is critical, requires an international effort, and will lead to much improved efficiency in neuroscience research. We call for the neuroscience community to join this standardisation effort.

Neuroscience is a multifactorial discipline where significant advances are made by combining theoretical, computational, experimental and technological approaches. The challenges of understanding function and dysfunction of the brain are still of unknown complexity and far from being met. Progress in therapeutics and clinical impact lags technical advances (Kapur et al., 2012), despite increased depth and scope of investigations. Results are currently published at an unprecedented rate, and for data and methods, stricter requirements are in motion toward more rigorous practices at funding agencies (NOT-OD-21–013) and most journals. Even so, there remain concerns about the large amount of poorly reproducible results (Ioannidis, 2007; Ioannidis et al., 2014; Baker, 2016). This represents a dire ‘lack of efficiency’ of neuroscience research amongst other fields (Chu & Evans, 2021).

To achieve the goals of basic and applied neuroscience, these fields require systematic, standardized and well-defined data organization practices and proper data description for effective content discovery and reproducibility. Deep understanding of the field necessitates 1) leveraging and extending existing theoretical frameworks and models for making testable predictions; and 2) probing experimental results and their interpretation by reanalysing data from different, new angles using cutting-edge analytic techniques. Given the complexity, scale, and multidisciplinary nature of the problem of understanding brain function, we argue such methods and practices for developing, integrating and testing theories and models must become radically more efficient, to keep up with the dramatic (and accelerating) advances in data acquisition.

There are evident reasons for the present state of practices in data sharing and management, not the least of which is the complexity of the nervous system itself. It is this profound complexity that requires the neuroscience research enterprise to efficiently integrate a broad set of results across manifold subfields. While these different subfields have common core concepts (like ‘function’, ‘experiment’, ‘observation’, ‘conclusion’, etc.), the data types, formats, experimental paradigms and appropriate metadata for these subfields differ, making integration of data and the development of a coherent theory of neural function a formidable challenge. Integration is also hampered by the culture of neuroscience (Ascoli, 2006) that still mostly values text-based articles over publishing dynamically usable research products, such as datasets with access methods, or Web based computational notebooks (Jupyter notebooks or Elife’s ‘Executable Research Articles’, Elife 2020). To achieve a more expedient understanding of brain function will require us to move beyond the present code and data archiving and sharing practices (Gleeson et al., 2017), information architectures, and publication models.

Is this problem resolving or compounding? Nationally and internationally, large investments in big data neuroscience initiatives are being undertaken (eHBP^1^, US BRAIN^2^, ENIGMA^3^, the Human Connectome Project (Elam et al., 2021), the China Brain Project, the Japanese Brain/MINDS project^4^, among others). A myriad of smaller, investigator-initiated research projects are continuously adding to our knowledge base, and individual investigators are thinking more broadly through extended collaborations. With this avalanche of data already underway, there are in fact few efforts to coordinate across communities the development of data standards and knowledge representation resulting from experimental research. It is well known that research incentive and funding structures are better suited for individual projects and initiatives but do not always foster collaboration in addressing the data and knowledge management in the bigger picture. Major funding of large scale consortia still prioritize new data generation over data integration, interoperability, management, or maximizing knowledge across existing databases. Efforts such as the NIH Common Fund's Stimulating Peripheral Activity to Relieve Conditions (SPARC) program and the BRAIN Initiative’s Cell Census Network (BICCN) in the US are reshaping these consortial practices but remain limited.

The funding bias towards new data but limited integration or curation introduces inefficiencies due to duplicated efforts, resulting in missing and redundant information, dramatically reducing the overall return on the research investment in the future. It is hard to quantify, but there is certainly a huge waste with poor reusability (Fergusson et al., 2014).

As neuroscience is an international effort, the effort to develop and implement standards is necessarily international in scope. Local efforts based on a few laboratories are unlikely to gather the critical mass necessary for adoption of a standard and the development of its ecosystem (tooling, training, etc.). Further, standards and repositories that are successful in supporting the aggregation of data across borders need to be sustained to be useful. In this way the development and adoption of rigorous and open data standards is seen as one of the key elements to promoting efficient collaboration and reuse. Effective data standards are tightly coupled with the availability of software tools which manage input and output data representations and transformations. For researchers remotely working in different subfields of neuroscience, improved standards and associated metadata is the only practical solution for efficient reuse of information to bridge across subfield domains (scales, species, cell type, resolution, brain functions, etc.). Funders and researchers are better at ‘developing’ than at ‘sustaining’, as there are less research incentives around maintenance of sustainable and coordinated information management infrastructure.

Is neuroscience a FAIR discipline, where data and results are Findable, Accessible, Interoperable and Reusable (Wilkinson et al., 2016)? FAIR is a set of increasingly accepted guiding principles for organizing and communicating the results of science so that they are understandable to both humans *and machines*. By contrast, current communication of results is still largely based on text (pdf—html) format articles. The articles themselves are findable, and often accessible (thanks to open initiatives like PubMed Central), but the central elements leading to the conclusions of the research (namely the data, software, detailed methods and complete results), are rarely FAIR. This lack of availability and transparency are at least partially causative of the problems that have emerged in terms of the reliability, reusability and reproducibility of the current research findings. There is a growing trend to meta-analyse sets of published data, but these will miss most data, i.e., studies for which data are not accessible or sufficiently reusable because of their format. Such omissions actually bias these important attempts at trans-study synthesis. Enabling the FAIR principles across the complete research workspace would make information aggregation feasible, efficient, and un- or less- biased (Mueller et al., 2018).

Today, the Web and other communication technologies provide fundamental tools to resolve this information integration issue. However, if our goal is to work efficiently and collaboratively and communicate research findings beyond exchanging papers, we need to establish a broader set of standards of communication. Anecdotally, the World Wide Web is successful because every browser “speaks” the same standards: http/html. Imagine how inefficient a Web search would be if 5 different browsers were required to execute separate search elements and we then had to manually integrate the results. The efficiency of Web search today is due to the international standardisation efforts and oversight of the World Wide Web Consortium (W3C). The inefficiency of a 5 browser manually-integrated search is suggestive of the current state of the art for a neuroscience query (i.e., “what is the cell density in brain regions associated with socialization, that expresses BDNF in the second trimester of development?”). Standards of data description and communication will also be necessary for machine and deep learning technologies to operate efficiently on large and diverse datasets, and reduce the huge current curation burden. Machines will need to extract standardized metadata for analyses to be efficient and unbiased.

So where do we go from here? A number of actions can be envisioned. One step is that national funding agencies should invest in international organizations and initiatives whose mission is focused on standardisation and education in neuroscience. The most established and experienced organization for neuroscience is the *International Neuroinformatics Coordinating Facility (INCF)*^5^, with its current network of 18 affiliated nations^6^. Through its new membership model, community-driven scientific interest groups, and international governance by members' representatives and stakeholders, the INCF provides the scaffolding and networking for community engagement around standards. A core mission of the INCF is to ensure that neuroscience is served by a set of well supported, non-overlapping standards that are easy to access and understand. The INCF has taken on the important role of acting as a standards organization for neuroscience, where standards and best practices can be reviewed, vetted and promoted (Abrams et al., 2021). Through this process, the INCF is creating a portfolio of standards that serve neuroscience and is developing training materials on their use. As the recognition by funders (such as NIH, NSF, Kavli, John and Laura Arnold Foundation, etc.), and the broader neuroscience community that this work is critical grows, the experience collected by INCF is unique and should be leveraged. Other international efforts include the International Brain Initiative, a consortium of the large international brain projects, which has recently established the Standards and Data Sharing Working Group to help achieve coordination with the INCF across large scale projects^7^. The IEEE has several standards efforts underway for neurotechnology^8^.

The successful development and adoption of standards will rely on the symbiotic development of current and novel tools that use and profit from these standards. The time frame for developing these standards and tools through community involvement, especially across international lines, is typically much longer than the grant cycles that power individual research programs. Thanks to investments and the work of dedicated volunteers working through organizations like INCF, a set of standards supporting neuroscience are starting to gain traction. A remarkable example of a successful standard is the “Brain Imaging Data Structure”^9^, for MRI data, started at an INCF meeting at Stanford, along with the development of many analysis tools that rely on the standard to automatically extract data and metadata (Gorgolewski et al., 2016, 2017). A substantial community has grown around the BIDS standard, with community-led extensions to domains such as magnetoencephalography (Niso et al., 2019) and electroencephalography (Pernet et al., 2019). The community has developed a formal governance procedure for extensions to the protocol, as well as a governance structure with an elected steering group. A second success is the Waxholm Space, a 3D MRI-based coordinate space for registering data in rat and mouse to a common coordinate system (Johnson et al., 2010; Okamura-Oho et al., 2012; Papp et al., 2014), adopted by the HBP and E-Brains. The Neurodata Without Borders (NWB) is an emerging standard for physiological data that has just issued its second version and is seeing uptake in the US BRAIN Initiative and other collaborative projects (Rübel et al., 2021). Finally, the US BRAIN Initiative is actively investing in the creation of new standards to support neuroscience^10^.

Programs for standardisation and promoting FAIR practices could also be set up through INCF or through scientific societies. These societies themselves, such as the Society for Neuroscience, the Organization for Human Brain Mapping and the clinical neurophysiology societies, can play a crucial role in encouraging standards development (e.g. Nichols et al., 2017), particularly as a growing number of journals and funding agencies are requiring deposition of code and data in a form suitable for secondary analyses. However, these organizations would need to form alliances and put in place the required funding tools as well as a vetting process, while such a process is already provided with the INCF.

Expanded information architecture and new software tools also have their role to play. The Open Connectome Project has enhanced the FAIRness of several prominent neuroscience studies (Vogelstein et al., 2018). These datasets are stored in a precomputed format in a publicly accessible cloud repository, and can be read, written, and viewed with nothing more than an Internet connection and browser (Charles et al., 2020). The Jupyter project (Kluyver et al., 2016) also proposes formats and infrastructure for data reuse. Efforts such as the European Human Brain Project (www.human brain project.eu) have made significant progress in knowledge graph architecture for neuroscience through projects such as the EBRAINS Knowledge Graph, a multi-modal metadata repository and query engine supporting experimental data and neuroscience data research. The SPARC Project has adopted FAIR principles in its data portal and knowledge graph, including full support for data citation (Osanlouy et al., 2021).

The FAIR data community and the INCF have learned -and continue to learn- some lessons from the decades old open source software community and standard organizations. Standard practice in open source software packages includes one line installation commands, and a quick-start tutorial, along with thoroughly documented code (Glatard et al., 2018; Vogelstein, 2018). These standards could translate to data stewardship practice in the form of brief readmes describing how to download and access the data, accompanied by more detailed metadata that is tethered to the data itself.

There have been multiple efforts to move neuroscience into e-Neuroscience through the development of standards and tools, some have been successful and some have not. However, we should refrain from interpreting the difficulties encountered as an argument against addressing the urgent requirements of a transformed and rapidly evolving field of neuroscience. Our technologies improve continuously, and empirical advantages, social pressures, and institutional policies have moved scientific communities towards open, data-driven and networked science. Recall also that the Web evolved in several phases, and standardized Web browsers were not a part of its early phase. Neuroscience is unlikely to ever be served by a single large database, but over the years, a functioning infrastructure comprising multiple databases and data repositories has emerged for sharing neuroscience data (Ascoli et al., 2017). We have today an opportunity to develop the necessary technology and make these infrastructures interoperable. We also have learned from the past about the technological and sociological barriers and are now in a better position to address them. The impact of increasing the reusability and therefore efficiency of neuroscience would be widespread and world-changing.

As the field of computational neuroscience evolves with new data acquisition methods, new hardware capabilities, and new analysis techniques, data standards will inevitably need to be updated or replaced. This reinforces the need for organizations to overlook these evolutions and the need for open governance as implemented by the INCF.

As scientists, we should pledge to work on the definition, development and implementation of standards and to foster a spirit of collaborative work in these developments. This work is not easy and will require dedication and support. We should ensure in our own research proposals that some effort will be set aside for the development of reproducible and FAIR research objects through international coordination. Ultimately research across the world is a *collective and collaborative* enterprise. Along with societies and funding agencies, individual scientists should take a proactive role in the evolution of this new world culture of FAIR neuroscience. One simple and concrete action is to participate in these global standardisation and coordination efforts with the INCF and together build a roadmap for FAIR neuroscience.

## Footnotes

1 https://www.humanbrainproject.eu/

2 https://www.braininitiative.nih.gov/index.htm

3 http://enigma.ini.usc.edu

4 https://en.wikipedia.org/wiki/Brain/MINDS

5 https://www.incf.org/

6 https://www.incf.org/about-us/who-we-are

7 https://www.internationalbraininitiative.org/

8 https://standards.ieee.org/industry-connections/neurotechnologies-for-brain-machine-interfacing.html

9 http://bids.neuroimaging.io/

10 https://grants.nih.gov/grants/guide/rfa-files/rfa-mh-19-146.html
